# Voltage threshold adjustments for a novel pulsed-field ablation catheter with integrated mapping capabilities: lessons from a case series

**DOI:** 10.1093/ehjcr/ytaf231

**Published:** 2025-05-12

**Authors:** Thomas Kueffer, Ajay Panakal, Claudia Herrera, Hildegard Tanner, Helge Servatius, Laurent Roten, Tobias Reichlin

**Affiliations:** Department of Cardiology, Inselspital, Bern University Hospital, University of Bern, Freiburgstrasse, 3600 Bern, Switzerland; Johnson & Johnson MedTech, Switzerland; Department of Cardiology, Inselspital, Bern University Hospital, University of Bern, Freiburgstrasse, 3600 Bern, Switzerland; Department of Cardiology, Inselspital, Bern University Hospital, University of Bern, Freiburgstrasse, 3600 Bern, Switzerland; Department of Cardiology, Inselspital, Bern University Hospital, University of Bern, Freiburgstrasse, 3600 Bern, Switzerland; Department of Cardiology, Inselspital, Bern University Hospital, University of Bern, Freiburgstrasse, 3600 Bern, Switzerland; Department of Cardiology, Inselspital, Bern University Hospital, University of Bern, Freiburgstrasse, 3600 Bern, Switzerland

**Keywords:** Voltage thresholds, Pulmonary vein isolation, Electroanatomical mapping, Case report

## Abstract

**Background:**

Accurate electroanatomical mapping relies on voltage thresholds to differentiate electrically inactive areas, fibrotic scar, and healthy myocardium. These thresholds have been well established for high-density mapping catheters with small, closely spaced electrodes. However, the optimal voltage thresholds for a novel pulsed-field ablation catheter with integrated mapping capabilities remain unclear. This case series evaluates different voltage thresholds for the variable-loop circular catheter (VLCC, Varipulse, Biosense Webster) compared with a dedicated high-density mapping catheter (Octaray, Biosense Webster).

**Case summary:**

Four patients undergoing left atrial catheter ablation—including pulmonary vein isolation (PVI), posterior wall ablation, and ablation for scar-related atrial flutter—were mapped using both the VLCC and Octaray catheter. The key findings include: (i) standard voltage thresholds for high-density catheters overestimate voltage in scarred and ablated tissue when applied to the VLCC, necessitating adjusted voltage settings; (ii) the VLCC effectively identified PVI and reconnections, posterior wall isolation, anterior wall scarring, and atrial flutter circuits; and (iii) while the VLCC identified areas of scar, its representation remained less precise compared with high-density mapping.

**Discussion:**

This case series demonstrates that the VLCC provides satisfactory mapping performance in common use cases but requires voltage threshold adjustments for accurate visualization. Despite its ability to detect ablation endpoints, scar characterization remains less accurate. Further quantitative analysis of electrogram differences and a prospective evaluation in a larger patient population are necessary to determine the optimal voltage thresholds for this catheter.

**Learning points**

- Voltage thresholds for the variable-loop circular catheter (VLCC) require adjustment to achieve visual parity with maps from high-density catheters.
- Despite larger electrode spacing, the VLCC accurately identifies pulmonary vein isolation status and arrhythmic substrates, though scar representation needs further refinement.

## Introduction

**Figure 1:**
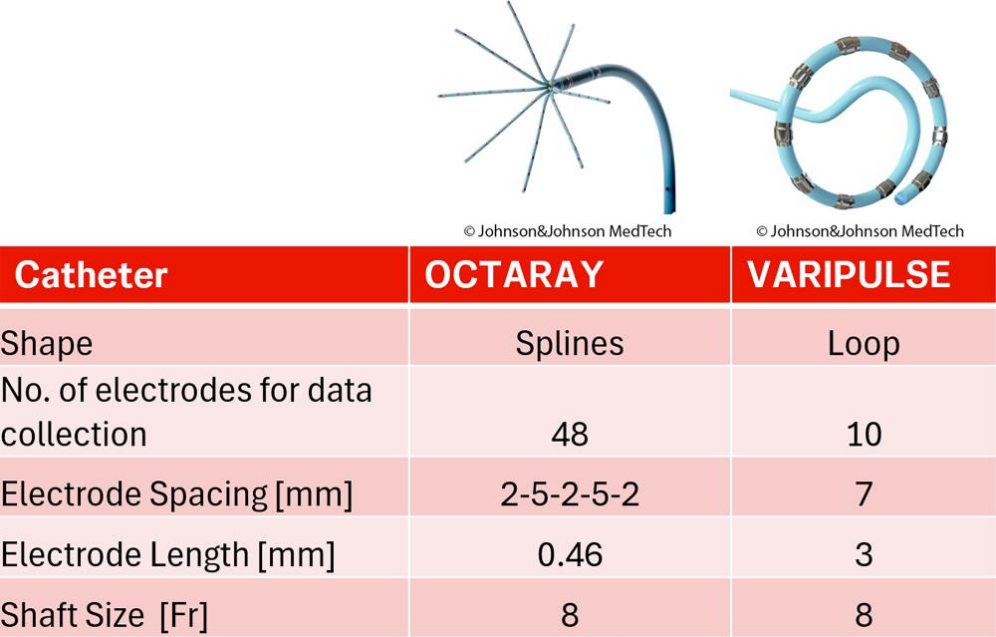
Comparison of a high-density mapping catheter (Octaray) and a variable-loop circular catheter (Varipulse). The larger electrode size and greater inter-electrode spacing of the VLCC influence signal amplitude and morphology. As a result, voltage threshold adjustments are necessary to achieve a mapping performance comparable to that of a high-density mapping catheter.

Voltage thresholds used to differentiate electrically inactive areas, fibrotic scar, and healthy myocardium are crucial for interpreting 3D electroanatomical maps. These thresholds have been established for high-density mapping catheters, which have small, closely spaced electrodes.^1^ However, both electrode size and inter-electrode distance can significantly affect the shape and amplitude of the recorded electrograms.^2^ As a result, each catheter requires evaluation of its own specific upper and lower voltage thresholds to accurately distinguish between inactive myocardium, potentially arrhythmogenic low voltage substrate, and healthy tissue (*Figure 1*).

A novel pulsed-field ablation (PFA) catheter system with full integration in a dedicated 3D electroanatomical mapping (3D-EAM) system has been recently introduced. It consists of an open-irrigated variable-loop circular catheter (VLCC) with 10 electrodes (electrode length 3 mm, inter-electrode spacing 7 mm center-to-center) and is capable of both mapping and PFA (Varipulse, Biosense Webster, Irvine, CA, USA). While its effectiveness for pulmonary vein isolation (PVI) has been shown previously, its mapping capabilities remain largely underexplored.^3,4^

### Case #1

**Figure 2:**
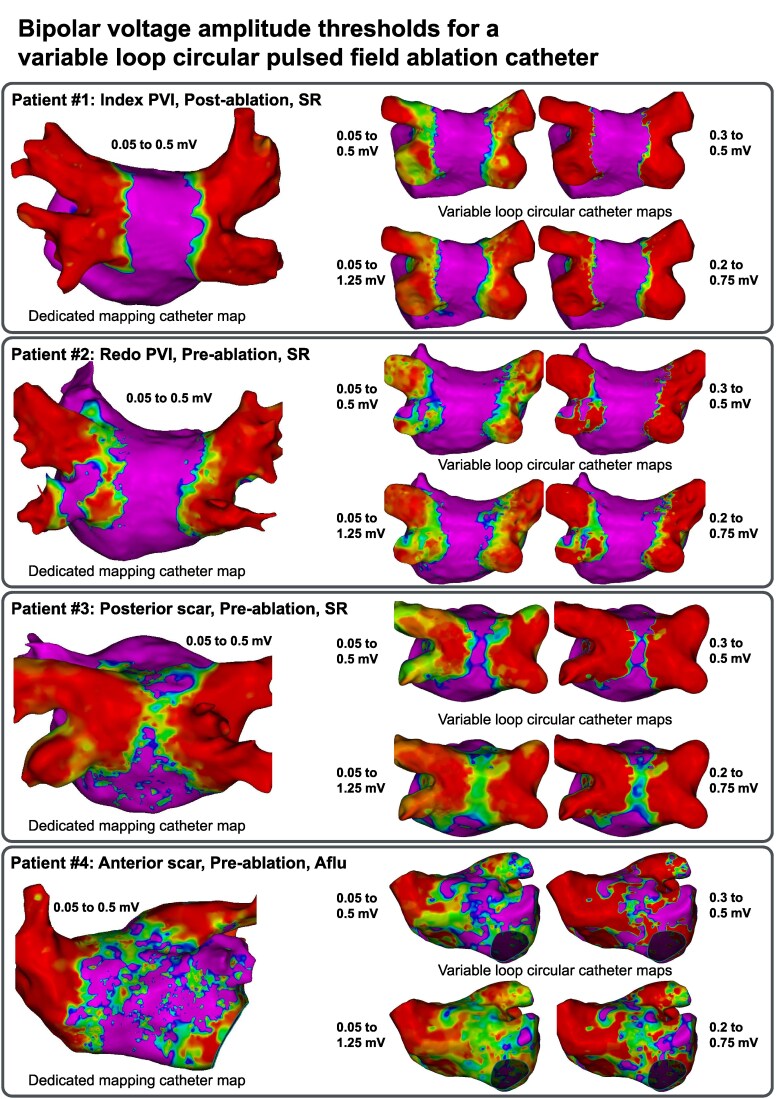
The left figure in each panel shows the high-density bipolar voltage map acquired with a dedicated mapping catheter (Octaray) and displayed with the standard voltage thresholds. The right figures show the maps acquired with the novel variable-loop circular catheter (VLCC, Varipulse) displayed with different bipolar voltage thresholds. Patient #1 underwent an index PVI with four isolated veins after PFA. Patient #2 underwent a redo PVI with a reconnected left lower pulmonary vein at the beginning of the procedure. Patient #3 shows wide antral PVI and scar on the posterior wall during a redo case. Patient #4 presented with an anterior scar. This map was acquired during a roof-dependent flutter, the tachycardia mechanism of which was also correctly identified by the VLCC. PFA, pulsed-field ablation; PVI, pulmonary vein isolation.

A 43-year-old male patient with symptomatic paroxysmal atrial fibrillation (AF) was referred for catheter ablation. Before the procedure, the patient underwent transoesophageal echocardiography and computed tomography to exclude intracardiac thrombi and to assess left atrial anatomy. Deep conscious sedation using midazolam, fentanyl, and propofol was used. Left atrium access was obtained after fluoroscopy-guided transseptal puncture using the Vizigo sheath (Biosense Webster). Pulmonary vein isolation was performed using the VLCC. Following ablation, 3D-EAM of the left atrium was performed to verify the endpoint of PVI during sinus rhythm. Subsequently, the 3D-EAM was repeated with a dedicated high-density mapping catheter with 48 electrodes (electrode length 0.5 mm, inter-electrode spacing 2 mm center-to-center, Octaray, Biosense Webster), using the same resolution and fill & colour threshold settings. Both maps showed isolation of all four pulmonary veins, and the acquired maps are displayed in *Figure 2*, Patient #1.

### Case #2

A 48-year-old male patient with persistent AF had undergone PVI using a cryoballoon ablation system. After the first ablation, he regressed to paroxysmal AF, but was still symptomatic from his episodes, and hence referred for a second ablation. Pre-procedural work-up and procedural setup was identical to Patient #1. Following transseptal puncture, 3D-EAM of the left atrium was performed during sinus rhythm using the VLCC. Subsequently, the 3D-EAM was repeated with a dedicated high-density mapping catheter (Octaray, Biosense Webster). Both maps indicated reconnection of the left lower pulmonary vein (*Figure 2*, Patient #2). Re-isolation of the left lower pulmonary vein was performed using the VLCC.

### Case #3

A 54-year-old female patient with paroxysmal AF had undergone PVI using a pentaspline PFA catheter (Farapulse, Boston Scientific). After the first ablation, she continued to have symptomatic AF episodes, and hence referred for a second ablation. Pre-procedural work-up and procedural setup was identical to Patient #1. Following transseptal puncture, 3D-EAM of the left atrium was performed in sinus rhythm using the VLCC. Subsequently, the 3D-EAM was repeated with a dedicated high-density mapping catheter (Octaray, Biosense Webster). Both maps indicated durable isolation of all four veins. The lesions, however, were extending quite far into the posterior wall leaving only a small channel of viable myocardium in between (*Figure 2*, Patient #3). Posterior wall ablation was performed using the VLCC.

### Case #4

**Figure 3:**
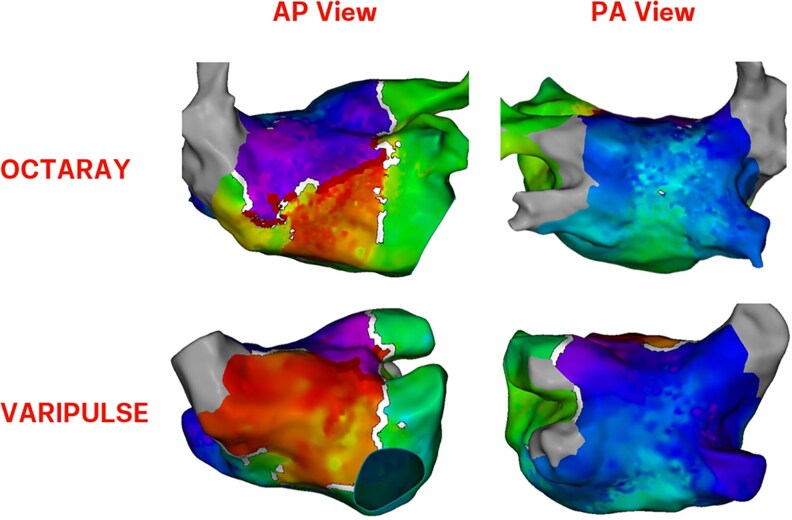
Local activation time maps of the roof-dependent flutter of Patient #4. This macro-reentrant circuit was well-identifiable using the variable-loop circular catheter.

A 77-year-old male patient with persistent AF had undergone PVI using a pentaspline PFA catheter (Farapulse, Boston Scientific). Eight months after the first ablation, he developed an atypical flutter and was referred for a second ablation. Pre-procedural work-up and procedural setup was identical to Patient #1. Following transseptal puncture, 3D-EAM of the left atrium was performed in atrial flutter using the VLCC. Subsequently, the 3D-EAM was repeated with a dedicated high-density mapping catheter (Octaray, Biosense Webster). Both activation maps correctly identified a roof-dependent atrial flutter; the voltage maps showed significant areas with low voltage scar at the atrial septum (*Figure 2*, Patient #4, *Figure 3*). The VLCC was used for ablation of the posterior wall, which terminated the atrial flutter.

## Discussion

Key observations of our case series (*Table 1*, *Figure 2*) include: (i) The larger inter-electrode distance of the VLCC results in different bipolar voltage amplitudes. Using the standard voltage thresholds for dedicated high-density mapping catheters causes overestimation of voltage in scarred and ablated tissue. This issue can be mitigated by changing both voltage thresholds. (2) Despite the larger electrode length and inter-electrode spacing of the VLCC, it correctly identified isolated and reconnected pulmonary veins (Patients #1 and #2), scar on the posterior wall during a redo case (Patient #3) and scar at the septum (Patient #4). Additionally, the tachycardia mechanism of the ongoing atypical left atrial flutter was correctly identified in the latter patient. (3) While the scarred area in Patient #4 was identified by the VLCC, none of the proposed threshold results in an accurate visual representation of the scar, as evidenced by high-density mapping.

**Table 1:** Patient overview

Patient	Age	Sex	Arrhythmias	Previous left atrial ablations	Lesion set	Octaray mapping points	Varipulse mapping points	Octaray mapping time (min)	Varipulse mapping time (min)
#1	43	M	Paroxysmal AF	None	PVI	1354	1645	11.9	8.5 min
#2	48	M	Persistent AF	1× PVI	Re-PVI + PWA	5748	5109	7.2	18.8 min
#3	54	F	Paroxysmal AF	None	PVI + PWA	3862	1737	9.4	7.2 min
#4	77	M	Persistent, Atypical flutter	1× PVI	Re-PVI + Anterior line	11 514	2977	7.4	18.3 min

PWA, posterior wall ablation.

Inherent to the nature of a case series, our findings are based on observations from selected cases. The VLCC’s ideal voltage thresholds and its accuracy for PVI verification have to be determined in a prospective comparison in a larger patient population. Based on initial visual assessment in these selected cases, a lower threshold of 0.2 mV and an upper threshold of 1.0 mV appear to provide the closest visual match to the high-density maps. For substrate characterization, a quantitative analysis is required, exploring the electrogram differences between the catheters in scarred areas.

In conclusion, this case series shows that the mapping performance of the novel VLCC was satisfying in common mapping use cases. However, adjustments of the voltage thresholds are required to obtain bipolar voltage maps that are visually comparable to maps acquired with a dedicated high-density mapping catheter.

## Lead author biography


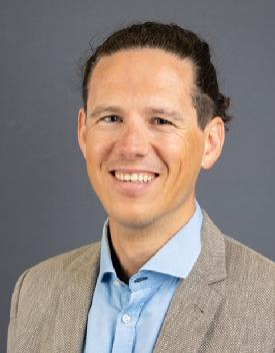


Thomas Kueffer, PhD, is a researcher specializing in cardiac arrhythmia treatment, focusing on pulsed-field ablation (PFA) and translational research to advance innovative therapies and improve clinical outcomes. His work bridges cutting-edge technology with practical applications in electrophysiology.

## Consent

All patients provided informed consent for participation in this study. This research complies with the COPE (Committee on Publication Ethics) guidelines.

## Data Availability

The data that support the findings of this study are available from the corresponding author upon reasonable request.
